# Application and progress of non-invasive imaging in predicting lung invasive non-mucinous adenocarcinoma under the new IASLC grading guidelines

**DOI:** 10.1186/s13244-024-01877-4

**Published:** 2025-01-02

**Authors:** Jinbao Feng, Xiaonan Shao, Jianxiong Gao, Xinyu Ge, Yan Sun, Yunmei Shi, Yuetao Wang, Rong Niu

**Affiliations:** Department of Nuclear Medicine, The Third Affiliated Hospital of Soochow University, The First People′s Hospital of Changzhou, Institute of Clinical Translation of Nuclear Medicine and Molecular Imaging, Soochow University, Changzhou Key Laboratory of Molecular Imaging, Changzhou, China

**Keywords:** Lung neoplasm, Invasive non-mucinous adenocarcinoma, IASLC grading, Imaging

## Abstract

**Abstract:**

Lung cancer is the leading cause of cancer-related deaths worldwide, with invasive non-mucinous adenocarcinoma (INMA) being the most common type and carrying a poor prognosis. In 2020, the International Association for the Study of Lung Cancer (IASLC) pathology committee proposed a new histological grading system, which offers more precise prognostic assessments by combining the proportions of major and high-grade histological patterns. Accurate identification of lung INMA grading is crucial for clinical diagnosis, treatment planning, and prognosis evaluation. Currently, non-invasive imaging methods (such as CT, PET/CT, and MRI) are increasingly being studied to predict the new grading of lung INMA, showing promising application prospects. This review outlines the establishment and prognostic efficiency of the new IASLC grading system, highlights the application and latest progress of non-invasive imaging techniques in predicting lung INMA grading, and discusses their role in personalized treatment of lung INMA and future research directions.

**Critical relevance statement:**

The new IASLC grading system has important prognostic implications for patients with lung invasive non-mucinous adenocarcinoma (INMA), and non-invasive imaging methods can be used to predict it, thereby improving patient prognoses.

**Key Points:**

- The new IASLC grading system more accurately prognosticates for patients with lung INMA.
- Preoperative prediction of the new grading is challenging because of the complexity of INMA subtypes.
- It is feasible to apply non-invasive imaging methods to predict the new IASLC grading system.

**Graphical Abstract:**

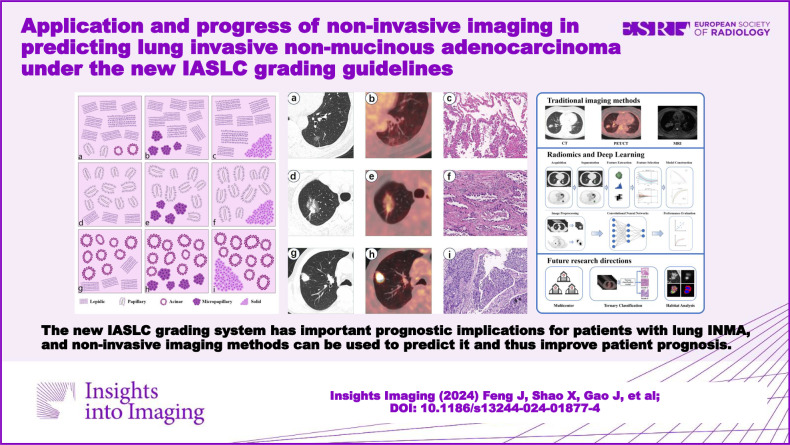

## Introduction

Lung cancer is the leading cause of cancer-related deaths worldwide, with its incidence and mortality rates topping those of all cancers in many countries [1–3]. Lung adenocarcinoma, the most common histological type, exhibits various histological patterns and proportions [4], showing significant heterogeneity in molecular characteristics, pathological presentation, prognosis, and treatment, with generally poor outcomes. About 70% of patients with lung adenocarcinoma are already at an advanced stage when diagnosed [5, 6]. Among all types of lung adenocarcinoma, invasive non-mucinous adenocarcinoma (INMA) is the most prevalent. Therefore, it is particularly important to accurately grade and diagnose them. However, previous grading systems have mostly relied on a single assessment of the predominant growth pattern, which is difficult to fully reflect the complexity of tumors and has insufficient correlation with the patient’s prognosis. To improve the prognosis of patients with lung INMA, in 2020, the pathology committee of the International Association for the Study of Lung Cancer (IASLC) proposed a new lung INMA grading system [7], which was adopted by the 2021 edition of the World Health Organization (WHO) classification of thoracic tumors [4]. This new system quantifies histological subtypes and their proportions in lung INMA, significantly improving the precision in patient prognostic stratification compared to previous grading systems focused on a single predominant growth pattern [8–10]. Accurate preoperative tumor grading is crucial in determining the treatment strategy for patients with lung INMA (including the scope of surgical resection, lymph node dissection methods, and whether to receive adjuvant therapy before and after surgery) [11–15]. Therefore, early identification and histological grading of lung INMA is important for optimizing patient management and achieving personalized precision diagnosis and treatment for lung cancer.

Due to the complexity of lung INMA subtypes, accurate diagnosis and grading have become a major challenge. Currently, definitive grading of lung INMA mainly relies on complete histological sampling of the tumor, while intraoperative rapid pathology and biopsy are invasive methods that cannot adequately assess the tumor growth pattern and grading [16]. Preoperative imaging examinations are important in predicting tumor malignancy, pathological subtypes, growth patterns, treatment efficacy, and prognosis [17–20]. Many studies have shown that non-invasive imaging techniques such as CT, PET/CT, and PET/MR can be used for lung cancer grading assessment [20–24]. Therefore, the development of non-invasive imaging methods has become a research hotspot to make up for the shortcomings of invasive methods. With the introduction of the IASLC grading system, more studies are exploring the feasibility of non-invasive imaging methods in predicting the new grading of lung INMA. Additionally, radiomics and deep learning methods have made significant progress in the field of lung cancer in recent years. These methods can extract high-throughput detailed features from medical images, which are difficult to distinguishable to the human, and have become important tools for disease diagnosis, treatment, and prognosis evaluation. Research has shown that radiomics and deep learning models are superior to conventional imaging feature models in predicting the new IASLC grading [25–28], demonstrating the promise and value of their application. This article will review the establishment and clinical value of the new IASLC grading system, summarize the current research status and progress in using non-invasive imaging methods to predict the new grading result, and suggest directions and challenges for future research to improve the prognosis of patients with lung INMA.

The literature search strategy is in the supplementary material.

## IASLC grading

### The establishment of IASLC grading

Since the 21st century, the classification of lung cancer has undergone three major adjustments (in 2004, 2015, and 2021) [4, 29, 30]. For lung adenocarcinoma, the most significant change occurred in 2011 with the joint release of a histological classification for lung adenocarcinoma by the IASLC, American Thoracic Society (ATS), and European Respiratory Society (ERS) [6]. This classification subdivided lung adenocarcinoma into atypical adenomatous hyperplasia (AAH), adenocarcinoma in situ (AIS), minimally invasive adenocarcinoma, invasive adenocarcinoma (IAC), and its variants, and further divided IAC into five major subtypes based on growth patterns: lepidic, acinar, papillary, micropapillary, and solid. In 2015, the WHO adopted this classification and renamed IAC to INMA, keeping the five growth patterns from the original classification [30, 31]. According to the 2011 IASLC/ATS/ERS classification and the 2015 WHO classification, lung INMA is divided into three differentiation grades based on the predominant growth pattern: low grade (predominantly lepidic), intermediate grade (predominantly acinar or papillary), and high grade (predominantly solid or micropapillary) [30], i.e., the Architectural (Arch) grading system, which can be used to predict the prognosis of lung adenocarcinoma patients [32–34]. However, although the Arch grading system provides useful prognostic information, it mainly focuses on the growth pattern of the tumor while neglecting minor or small amounts of high-grade patterns, which limits its prognostic accuracy. Even lung adenocarcinomas with small amounts of high-grade patterns, such as micropapillary or solid, generally have poor prognosis [35–38].

To more accurately assess the prognosis of lung INMA, the IASLC launched a new grading system in 2020 [7]. This system classifies tumors based on the proportions of the predominant histological pattern and high-grade patterns (with a cutoff of 20%) [7, 39], dividing lung INMA into three grades: Grade 1 (well-differentiated), predominantly lepidic with no or less than 20% high-grade components; Grade 2 (moderately differentiated), predominantly acinar or papillary with no or less than 20% high-grade components; Grade 3 (poorly differentiated), any histological pattern with 20% or more high-grade components. High-grade components include solid, micropapillary, and complex glandular structures (cribriform, fused glands, or single cells infiltrating desmoplastic stroma). This new grading system applies only to lung INMA, while AIS and AAH are classified as precursor glandular lesions. This grading system has been incorporated into the 5th edition (2021) of the WHO Classification of Thoracic Tumors, which no longer describes lung INMA simply as adenocarcinoma dominated by a specific histological pattern but recommends using the IASLC grading system [4]. A comparison of different grading systems for lung INMA is shown in Table 1.

**Table 1:** Comparison of different grading systems for lung INMA

Grading system	Level	Grading criteria
Sica’s grading 1 point (lepidic), 2 points (papillary or acinar), 3 points (solid or micropapillary), sum the scores of two main patterns	Low grade	3 points
	Intermediate grade	4 points
	High grade	5 or 6 points
Arch grading Based on the predominant histological growth pattern	Low grade	Predominantly lepidic
	Intermediate grade	Predominantly acinar or papillary
	High grade	Predominantly solid or micropapillary
IASLC grading Based on the combination of the predominant histological pattern and the proportion of high-grade patterns (cutoff 20%)	Grade 1	Predominantly lepidic, with < 20% high-grade components^a^
	Grade 2	Predominantly acinar or papillary, with < 20% high-grade components
	Grade 3	Any histological type with ≥ 20% high-grade components

^a^ High-grade components include solid, micropapillary, and complex glandular structures (including cribriform and other poorly formed glandular patterns)

### Clinical value of IASLC grading

Since the new IASLC grading system proposal, several studies have confirmed its significant practical value in predicting the prognosis of patients with lung INMA. Kagimoto et al [40] found that there were significant differences in recurrence-free survival (RFS) and overall survival (OS) among different grades. Specifically, patients reclassified from intermediate grade (Arch grading) to Grade 3 (IASLC grading) had a significantly worse prognosis (5-year RFS of 65.2%) compared to IASLC Grade 2 patients (5-year RFS of 77.1%). Additionally, Fujikawa et al [41] found that higher IASLC grades were associated with shorter OS and RFS, with Grade 3 being an independent prognostic factor [hazard ratio (HR) of 2.78 and 2.00, respectively]. She et al [42] further confirmed that the new IASLC grading system could be applied in routine pathological diagnosis and effectively stratified patient prognosis. The study by Deng et al [14] also supports this grading system as an effective tool for prognostic evaluation. The studies indicate that the new IASLC grading system can accurately predict patient prognosis, providing clinicians with important guidance for postoperative treatment decisions.

The histological grading of lung INMA is also closely related to pathological aggressiveness and mediastinal lymph node metastasis, playing a crucial role in determining surgical strategies for patients with clinical stage I lung adenocarcinoma. For example, high-grade adenocarcinomas usually require lobectomy and systematic lymph node dissection [11–13]. Additionally, the prognostic stratification of the new IASLC grading system can indicate whether patients need adjuvant chemotherapy. Hou et al [15] showed that patients reclassified from intermediate grade in the old system to IASLC Grade 3 could receive additional benefits from adjuvant chemotherapy. Furthermore, a study [41] indicates that the new grading of lung INMA is associated with genetic mutations. Compared to Grade 3 tumors, epidermal growth factor receptor mutations are more common in Grade 1 and 2 tumors (71.6%), while Kirsten rat sarcoma viral oncogene mutations and rearranged during transfection fusions are predominant in Grade 3 tumors (65.0% and 75.0%, respectively). This information is crucial for selecting targeted therapy or neoadjuvant chemoradiotherapy regimens for patients. Therefore, the new IASLC grading system is essential for accurately predicting lung INMA patients’ prognosis, significantly guiding individualized treatment plans, and improving patient outcomes.

### Comparative studies of IASLC grading

Since the release of the new IASLC grading system, many groups have conducted comparative studies on various lung adenocarcinoma grading systems. Rokutan-Kurata et al [8] conducted a comparison of the new IASLC grading system, the Arch system (based on the predominant pattern), and Sica’s system (based on the two main patterns) in a large cohort of 1241 patients. The study found that the predictive performance of the IASLC system for recurrence and death was comparable to that of the Sica’s system and slightly higher than that of the Arch system. This indicates that the IASLC and Sica’s systems can accurately reflect the prognosis of lung INMA patients, outperforming the Arch system. Yanagawa et al [9] showed that all three grading systems could be used to predict the prognosis of lung cancer patients. However, the Grade 3 tumors defined by the new IASLC grading system had a higher hazard ratio compared to Grade 1 tumors (OS: IASLC vs. Arch vs. Sica’s grading systems: HR = 3.77 vs. 3.03 vs. 2.63; RFS: HR = 4.25 vs. 2.69 vs. 2.4), indicating that the new IASLC grading system is superior in detecting high-grade malignancy. Additionally, Kagimoto et al [40] showed that patients reclassified from intermediate grade in the previous Arch system to IASLC Grade 3 had significantly worse prognosis compared to IASLC Grade 2 patients but not substantially different from high-grade or IASLC Grade 3 patients, further demonstrating the better prognostic stratification ability of the new IASLC system. Lucà et al [10] also confirmed the independent prognostic value of the IASLC grading system in lung INMA, which provided better prognostic grouping.

**Fig. 1:**
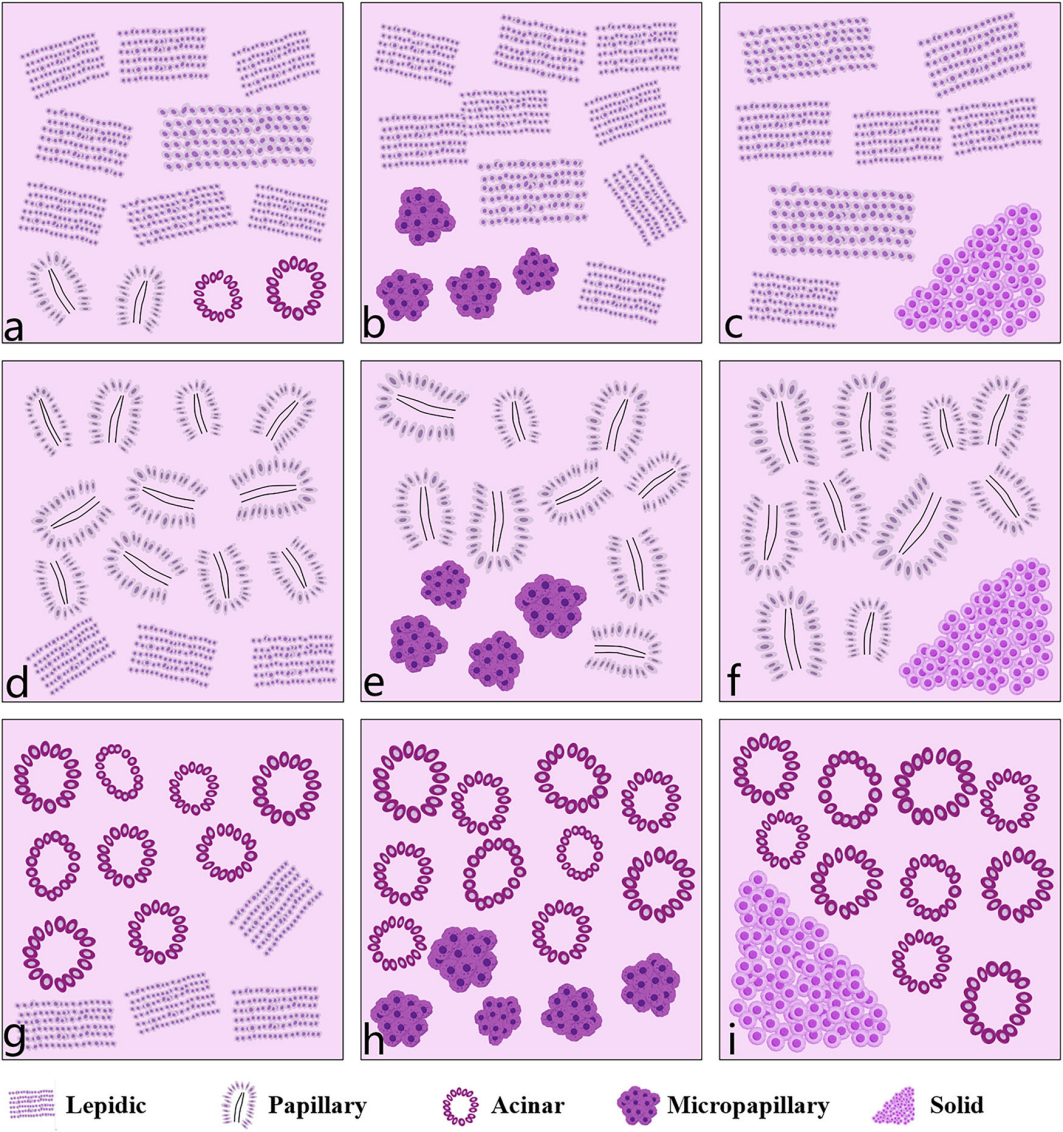
Pathologic schematic illustrations of the Arch grading system and the new IASLC grading system (By Figdraw). **a** The growth patterns of lung INMA in this case are predominantly lepidic with a little acinar and papillary, low grade in Arch grading system and grade 1 in the new IASLC grading system, both of which are well-differentiated. **b** Predominantly lepidic with micropapillary more than 20%, low grade (well-differentiated) in Arch grading system but grade 3 (poorly differentiated) in the new IASLC grading system. **c** Predominantly lepidic with solid more than 20%, low grade (well-differentiated) in Arch grading system but grade 3 (poorly differentiated) in the new IASLC grading system. **d** Predominantly papillary with a little lepidic, intermediate grade in Arch grading system and grade 2 in the new IASLC grading system, both of which are moderately differentiated. **e** Predominantly papillary with micropapillary more than 20%, intermediate grade (moderately differentiated) in Arch grading system but grade 3 (poorly differentiated) in the new IASLC grading system. **f** Predominantly papillary with solid more than 20%, intermediate grade (moderately differentiated) in Arch grading system but grade 3 (poorly differentiated) in the new IASLC grading system. **g** Predominantly acinar with a little lepidic intermediate grade in Arch grading system and grade 2 in the new IASLC grading system, both of which are moderately differentiated. **h** Predominantly acinar with micropapillary more than 20%, intermediate grade (moderately differentiated) in Arch grading system but grade 3 (poorly differentiated) in the new IASLC grading system. **i** Predominantly acinar with solid more than 20%, intermediate grade (moderately differentiated) in Arch grading system but grade 3 (poorly differentiated) in the new IASLC grading system

In summary, the new IASLC grading system significantly improves the accuracy of assessment of prognosis in lung INMA patients compared to previous grading systems. There are two main reasons for this: the new IASLC system includes new high-grade types in its grading criteria—complex glandular structures. Studies have shown that these complex glandular structures are associated with high mitotic rates, tumor necrosis, and pulmonary lymphovascular invasion, which are linked to poor prognosis [43–45]. Second, previous grading systems were based solely on the predominant growth pattern, whereas even a small amount of high-grade patterns can affect prognosis. The new IASLC grading system addresses the issue of not quantitatively considering the proportion of high-grade patterns in previous systems by defining any lung INMA with 20% or more high-grade patterns as Grade 3 or poorly differentiated INMA, whose clinical behavior and prognosis are similar to those of lung INMA dominated by high-grade patterns. Pathologic schematic illustrations of the Arch grading system and the new IASLC grading system are shown in Fig. 1.

## Prediction of IASLC grading based on non-invasive imaging

In current clinical practice, diagnosing lung INMA pathological tissue patterns and grading primarily rely on pathological specimens obtained from complete tumor resection. Even invasive biopsy techniques struggle to accurately assess tumor grading because of the limited tissue samples obtained [16]. Therefore, there is an urgent need for a non-invasive examination method. Since the 2011 IASLC/ATS/ERS histological classification of lung adenocarcinoma, numerous studies have begun using non-invasive imaging methods to predict the Arch grading, including predicting high-grade patterns alone. These studies are mainly based on CT, PET/CT, PET/MR images, and radiomics [18, 20–24]. Given the limitations of the Arch grading, the new IASLC grading system has been developed and gradually replaced the old grading system, gaining widespread acceptance and application. With the promotion of the new grading system, more researchers are exploring imaging methods and radiomics approaches to predict the new IASLC grading system, aiming to more accurately assess the pathological characteristics and prognosis of lung adenocarcinoma patients through these advanced imaging technologies. The results of these studies are summarized in Table 2. And comparison of every method in predicting the new IASLC grading system is in Table 3.

**Table 2:** Studies on imaging methods for predicting the new IASLC grading of lung INMA

Author	Year	Sample size	Imaging methods	Number of imaging features	AUC
Volmonen et al [51]	2022	162 Grade 1: 48 Grade 2: 48 Grade 3: 66	CT	Distinguish Grade 2 from 1: 4 CT semantic features; Distinguish Grade 3 from 2: 3 CT semantic features	Distinguish Grade 2 from 1: AUC = 0.783; Distinguish Grade 3 from 2: AUC = 0.759
Liang et al [53]	2023	379 Grade 1: 57 Grade 2: 233 Grade 3: 89	CT	3 CT semantic features	Predict Grade 3: AUC = 0.907
Liu et al [55]	2023	302 Grade 1: 81 Grade 2: 203 Grade 3: 18	CT	1 semantic CT feature	Predict Grade 2/3: Training set: AUC = 0.890; Test set: AUC = 0.832
Zhang et al [56]	2022	76 Grade 1: 3 Grade 2: 43 Grade 3: 30	Spectral CT	2 CT semantic features	Predict Grade 3: AUC = 0.652–0.688
Fujikawa et al [41]	2022	781 Grade 1: 114 Grade 2: 438 Grade 3: 229	PET/CT	1 PET semantic feature	Predict Grade 3: AUC = 0.828
Yang et al [52]	2023	227 Grade 1 and Grade 2: 167 Grade 3: 60	PET/CT	2 clinical features; 2 CT semantic features; 1 PET semantic feature;	Predict Grade 3: AUC = 0.869
Dang et al [75]	2024	153 Grade 1: 18 Grade 2: 85 Grade 3: 50	MRI	1 clinical feature; 1 CT semantic feature; 2 MRI semantic features	Predict Grade 3: AUC = 0.778
Yang et al [25]	2023	303 Grade 1 and Grade 2: 173 Grade 3: 130	CT-based radiomics	9 CT radiomic features 4 CT semantic features	Predict Grade 3: Training set: AUC = 0.915; Test set: AUC = 0.838
Li et al [26]	2023	682 Grade 1: 250 Grade 2: 353 Grade 3: 79	CT-based radiomics	6 CT radiomic features	Validation set: Grade 1 vs 2/3: AUC = 0.900; Grade 1/2 vs 3: AUC = 0.929
Pei et al [27]	2023	350 Grade 1: 37 Grade 2: 296 Grade 3: 17	CT-based deep learning combined with radiomics	40 CT radiomic features	Average AUC of predicting Grades 1, 2, and 3: Internal cohort: AUC = 0.928; Independent test set: AUC = 0.837; External test set: AUC = 0.748
Zhong [28]	2023	2638 Grade 1: 403 Grade 2: 1281 Grade 3: 954	PET/CT-based deep learning	-	Predict Grade 3: Validation set: AUC = 0.862; External cohort: AUC = 0.844; Prospective cohort: AUC = 0.851

**Table 3:** Comparison of non-invasive imaging techniques and radiomics methods in predicting the new IASLC grading system

Imaging methods	Predictors/models	Advantages	Shortcomings
**CT**	Air bronchogram Spiculation Vascular convergence Well-defined margins Pure solid appearance CTR CT value	Fast imaging Short inspection time High resolution Low radiation dose Wide range of applications	Some features are difficult to recognize or interpret Complex calculations may be required for certain parameters Dependent on physician experience and expertise Current relevant studies are predominantly single-center
**Spectral CT**	Arterial phase CT_60keV_ Venous phase CT_50keV_-CT_70keV_	Reduced radiation dose Improving the differentiation of tissue components Providing more accurate diagnostic information Acquisition of images for energy spectrum analysis without the need to pre-determine the lesion	Higher equipment costs Complex data processing Low popularity of current clinical applications Existing studies have only included solid lung adenocarcinoma and the sample size is small and unrepresentative
**PET/CT**	SUVmax CT features	It can not only show the morphological changes and pathological processes of tumors through CT imaging but also reveal the metabolic activities of tumors through PET imaging SUVmax is widely used and easy to access	Expensive Long examination time Relatively high radiation dose The correlation between other metabolic parameters (e.g., MTV and TLG, etc.) and the new IASLC classification is unclear
**MRI**	ADC T2CR	Radiation free T1W sequences can depict small lung nodules and capture their morphological characteristics T2W sequences can reveal the internal signal properties of the lesion DWI is useful in the differentiation of benign and malignant lung nodules and in the evaluation of lung cancer staging	Less used in lung disease Long and costly examination Less relevant research
**Radiomics**	Radiomic models	Applicable to various imaging techniques (e.g., CT, MRI, PET, etc.) Extracting a large number of quantitative features from medical images for early diagnosis, prognostic assessment, and treatment response monitoring Providing a new, non-invasive, and convenient way to develop treatment strategies by utilizing advanced image analysis tools and rapid medical imaging data processing	Need to acquire large-scale and high-quality medical image data The generalization performance of the model is affected by various factors More clinical validation is needed to establish their reliability and effectiveness
**Deep Learning**	Deep learning models	Powerful image recognition and feature extraction capabilities, which can improve diagnostic accuracy and early detection efficiency Automating the processing of large amounts of complex image data	Reliance on high-quality labeled data Poor interpretability of models Ethical and regulatory challenges that may be encountered when generalizing applications in clinical settings

*CTR* consolidation tumor ratio, *SUVmax* the maximum standardized uptake value, *MTV* metabolic tumor volume, *TLG* total lesion glycolysis, *ADC* apparent diffusion coefficient, *T2CR* T2-weighted contrast ratio, *T1W* T1-weighted, *T2W* T2-weighted, *DWI* diffusion-weighted imaging

### Prediction of IASLC grading based on CT imaging

CT imaging has the characteristics of fast, convenient and high resolution. With the widespread use of chest CT for lung cancer screening globally and advances in technology, early detection and timely treatment of lung cancer have become common, significantly reducing lung cancer mortality [46, 47]. Previous studies have shown that CT imaging features (such as air bronchogram, well-defined margins, and vascular convergence) can help identify different pathological histological patterns of lung adenocarcinoma and stratify patients [21, 48–50]. After introducing the new lung INMA grading system, some studies have analyzed the relationship between preoperative CT quantitative parameters and morphological features with IASLC grading. The survey by Volmonen et al [51] showed that air bronchograms decreased from Grade 1 to Grade 3 lung INMA while the consolidation tumor ratio (CTR) increased. In subgroup analysis, the solid/subsolid type, CTR, well-defined margins, and air bronchogram were independent factors distinguishing Grade 2 from Grade 1 lung INMA, the area under the curve (AUC) of this model was 0.783; the size of the consolidation part/whole tumor ratio, size of the consolidation part, and well-defined margins were independent factors distinguishing Grade 3 from Grade 2 lung INMA (model AUC = 0.759), similar to the findings of Yang et al [52]. Additionally, the study by Liang et al [53] found that higher CT values, CTR, and larger tumor size were independent risk factors for predicting Grade 3 lung INMA (model AUC = 0.907), and identified their thresholds in distinguishing Grade 1 from Grade 2 and Grade 2 from Grade 3 lung INMA, respectively: CT value: < −420 HU and ≥ −205 HU, CTR: < 25% and ≥ 75%, tumor size: < 12 mm and ≥ 17 mm. The study by Kawaguchi et al [54] indicated that pure solid appearance was more common in IASLC Grade 3 tumors compared to Grades 1 and 2, with pure solid appearance, round/oval shape, spiculation, and air bronchogram being independent predictors of Grade 3 lung INMA. Fujikawa et al [41] also showed that the proportion of ground-glass nodules decreased, while the proportion of solid nodules significantly increased with higher tumor grade. Liu et al [55] used computer-assisted and 3D methods to measure the solid component within the tumor, demonstrating that the consolidation/tumor ratio of volume at an attenuation threshold of −250 HU correlated well with lung INMA grading. Their model predicted high-risk (Grade 2/3) lung INMA with an AUC of 0.890 in the training set and 0.832 in the test set, outperforming 2D (training set AUC = 0.821, test set AUC = 0.760) and semantic models (training set AUC = 0.809, test set AUC = 0.710). However, there may be some limitations in the prediction of new IASLC grading based on CT imaging. On the one hand, CT images may be affected by anatomical structures and individual differences in patients, making certain features difficult to identify or interpret. The calculation of some CT quantitative parameters (e.g., CTR) may require complex technical support, which also limits its application in clinical routine. Second, interpreting CT images relies on the experience and expertise of physicians, which may lead to inconsistent results between different physicians. Finally, although some studies have shown good predictive performance, more clinical validation is needed to ensure its universality and accuracy in different populations and settings.

Additionally, Zhang et al [56] explored the diagnostic performance of spectral CT in preoperatively evaluating different pathological grading systems in 76 patients with solid lung INMA. The results indicated that in Stage I and II adenocarcinoma, the enhancement amplitude, k values, and CT_40keV_-CT_60keV_ during the arterial phase, as well as the enhancement amplitude, k values, and CT_40keV_-CT_70keV_ during the venous phase, exhibited high diagnostic efficiency in distinguishing high-grade lung adenocarcinoma according to the 2011 classification (AUC ranging from 0.700 to 0.853). However, for the high-grade (Grade 3) lung adenocarcinoma in 2020 classification, the diagnostic efficiency of arterial phase CT_60keV_ and venous phase CT_50keV_-CT_70keV_ was lower (AUC ranging from 0.652 to 0.688), suggesting that the parameters of spectral CT are slightly more effective for the 2011 high-grade group of lung adenocarcinoma compared to the 2020 version. As a novel spectral CT technology, the dual-layer detector spectral CT can simultaneously collect high and low-energy information during each routine scan, allowing for spectral analysis-ready image acquisition without pre-judging the lesion, thus having broad clinical application value [57]. However, the above study indicates that the predictive performance of spectral CT for the new IASLC grading system is less ideal, which may be related to the survey only including solid lung adenocarcinoma and not evaluating subsolid lung adenocarcinoma, along with the small sample size. Therefore, further research is needed to explore the potential application of spectral CT in the preoperative evaluation of the new grading system.

### Prediction of IASLC grading based on [^18^F]FDG PET/CT imaging

Fluorodeoxyglucose (FDG), a glucose analog, can be taken up by tumor cells, with its uptake increasing as the Warburg effect [58] intensifies. This metabolic activity is associated with cell differentiation, proliferation, gene expression, circulating tumor cells, tissue hypoxia, and patient prognosis. Therefore, [^18^F]FDG PET/CT imaging can reveal both the morphological changes of tumors through CT imaging and the metabolic activity of tumors through PET imaging. Currently, [^18^F]FDG PET/CT is widely used in diagnosing, staging, and therapeutic evaluating lung cancer [59–63]. Among its parameters, the maximum standardized uptake value (SUVmax) is crucial for quantifying glucose consumption by tumor cells and has been employed to predict the prognosis of non-small cell lung cancer [64]. Studies by Kawaguchi et al [54] and Jeon et al [37] indicated that the SUVmax of Grade 3 lung INMA was significantly higher than that of Grade 2 and Grade 1 lung INMA, with median SUVmax values of 4.7 vs. 2.0 vs. 1.3 and 5.3 vs. 2.2 vs. 1.5, respectively. Fujikawa et al [41] found that SUVmax significantly increased with tumor grade and was an independent predictor for Grade 3 lung INMA, with a cutoff value of 3.45, sensitivity of 80.5%, specificity of 74.1%, and an AUC of 0.828. Additionally, Yang et al [52] developed a model combining SUVmax with smoking history, carcinoembryonic antigen, CTR, and air bronchogram or vacuole sign, which could effectively predict Grade 3 lung INMA (AUC = 0.869), outperforming the use of SUVmax alone (AUC = 0.793). Current research consistently shows a close relationship between SUVmax and IASLC grading, yet a universally accepted standard for predicting IASLC grading using SUVmax has not been established. Different studies define varying cutoff values of SUVmax, and the potential nonlinear relationship between them remains unclear. Therefore, further research is needed to explore the association between SUVmax and the new IASLC grading system and to establish its validity as a predictive tool.

**Fig. 2:**
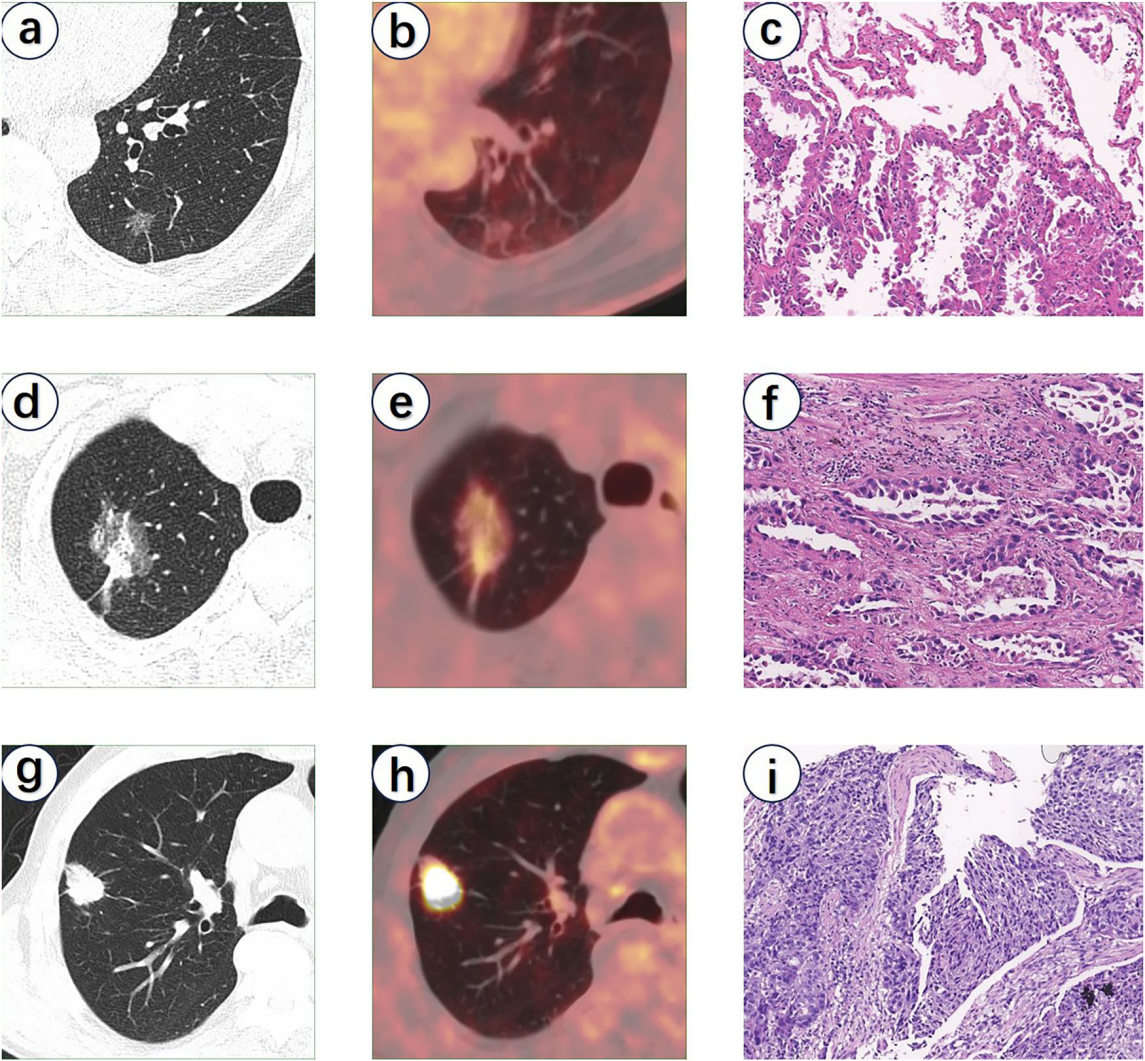
A 73-year-old man with grade 1 lung INMA (**a**–**c**). **a** A CT image showing 14 × 12 mm pure ground-glass nodule with air bronchogram, well-defined margin and strip shadow. **b** An [^18^F]FDG PET/CT image showing no fluorodeoxyglucose uptake (SUVmax = 1.7). **c** Photomicrograph (H and E, × 100) of grade 1 lung INMA. A 64-year-old woman with grade 2 lung INMA (**d**–**f**). **d** A CT image showing a 29 × 20 mm lobulated part-solid nodule with air bronchogram, spiculation and pleural traction. **e** An [^18^F]FDG PET/CT image showing mild fluorodeoxyglucose uptake (SUVmax = 3.6). **f** Photomicrograph (H and E, × 100) of grade 2 lung INMA. A 73-year-old woman with grade 3 lung INMA (**g**–**i**). **g** A CT image showing a 25 × 19 mm lobulated pure solid nodule with spiculation and pleural traction but without air bronchogram. **h** An [^18^F]FDG PET/CT image showing strong fluorodeoxyglucose uptake (SUVmax = 19.6). **i** Photomicrograph (H and E, × 100) of grade 3 lung INMA

Additionally, compared to the commonly used SUVmax, metabolic parameters such as metabolic tumor volume (MTV) and total lesion glycolysis (TLG) are important tools for evaluating tumor burden and aggressiveness. These parameters are frequently used to assess the overall metabolic activity of tumors and have significant implications for evaluating tumor prognosis [65, 66]. However, limited studies currently investigate the relationships between MTV, TLG, and the new IASLC grading system. Yang et al [52] demonstrated that TLG was closely associated with IASLC grading. The TLG for Grade 3 tumors (median value of 6.82) was significantly higher than for Grade 1 and 2 tumors (3.39). In contrast, no significant correlation was found between MTV and IASLC grading. Previous studies on the correlation between MTV, TLG, and the older grading systems have shown mixed results. Wang et al [67] reported that higher histological grades of lung adenocarcinoma were associated with higher MTV and TLG values. However, Chen et al [68] found that MTV and TLG were ineffective predictors of lung INMA’s pathological type and grade. Given that the new IASLC grading system supplements and updates the previous system based on predominant growth patterns, further comprehensive and systematic studies are needed to verify the relationship between PET-related metabolic parameters and the new grading system. Typical cases are shown in Fig. 2.

### Prediction of IASLC grading based on MRI

MRI technology has significantly advanced its application to lung diseases in recent years. T1-weighted (T1W) sequences can depict small lung nodules and capture their morphological characteristics [69–71], while T2-weighted (T2W) sequences can reveal the internal signal properties of lesions [72]. Additionally, diffusion-weighted imaging (DWI) has been shown to assist in distinguishing benign from malignant lung nodules and in the staging evaluation of lung cancer [73, 74]. In a previous study by Li et al [20] on the correlation between PET/MR and the Arch grading system, both DWI and diffusion kurtosis imaging demonstrated diagnostic value for the histological grading of lung adenocarcinoma. Recently, Dang et al [75] explored the utility of MRI in predicting the new IASLC grading in 153 lung INMA patients. The study found that the apparent diffusion coefficient (ADC) value of high-grade adenocarcinomas (Grade 3) was significantly lower than that of low-grade (Grade 1 and Grade 2) adenocarcinomas (AUC = 0.645), while the T2CR value of high-grade adenocarcinomas was higher than that of the low-grade group (AUC = 0.627). When combining T2-weighted contrast ratio (T2CR), ADC, gender, and maximum diameter, the diagnostic performance of the combined model was significantly enhanced (AUC = 0.778), indicating that MRI has the potential to become an effective tool for preliminary evaluation of the new IASLC grading of lung INMA. Although the use of MRI in lung diseases is limited due to its longer examination time and higher costs, which restricts its application, advances in medical imaging technology and improvements in healthcare conditions may eventually make MRI or PET/MR important non-invasive imaging methods for predicting the new IASLC grading.

### Prediction of IASLC grading based on radiomics and deep learning

Radiomics uses high-throughput technology to extract and analyze many advanced quantitative imaging features from medical images. By employing automated or semi-automated analytical methods, this approach converts imaging data into a high-resolution, analyzable data space [76]. Radiomics leverages advanced image analysis tools and rapid medical imaging data processing to provide a new, non-invasive, and convenient way to inform treatment strategies. This method improves the accuracy of diagnosis, prognosis, and prediction, optimizing clinical and economic benefits for patients [77]. With the introduction of the new IASLC grading system, studies using radiomics to build models and predict this grading system have been increasing. Yang et al [25] retrospectively analyzed clinical and CT imaging data from 303 lung INMA cases and developed a model based on nine radiomic features to generate a Radscore. They combined this with clinical-imaging features to construct a combined model for the preoperative prediction of tumors graded as Grade 3 by IASLC. The results showed that the diagnostic performance of both the combined model and the radiomic model was superior to that of the clinical-imaging features model alone in both the training and test sets (training set AUCs: 0.915 vs. 0.897 vs. 0.882; test set AUCs: 0.838 vs. 0.819 vs. 0.782). Li et al [26] developed a radiomic model based on low-dose CT (LDCT) that accurately predicted IASLC grading. The diagnostic performance of this model in the validation cohort (AUC for distinguishing Grade 1 vs. Grades 2/3 = 0.900; AUC for distinguishing Grades 1/2 vs. Grade 3 = 0.929) was comparable to that of radiology experts (AUC for distinguishing Grade 1 vs. Grades 2/3 = 0.840; AUC for distinguishing Grades 1/2 vs. Grade 3 = 0.852) and superior to the quantitative-semantic model and less experienced radiologists. These results indicate that the LDCT radiomic model can assist less experienced radiologists in stratifying the new IASLC grading in lung cancer screening.

Deep learning (DL) technology has become a valuable tool in disease diagnosis, treatment decision-making, and prognosis assessment in recent years. It allows for high-dimensional quantification of medical images and extraction of more detailed features than the human eyes [78, 79]. As an emerging pattern analysis method that automatically extracts complex data features at a high level of abstraction, DL models support incremental feature extraction without human intervention, demonstrating significant advantages over traditional radiomics methods [80]. Pei et al [27] developed a CT-based method combining DL and radiomics to provide a non-invasive IASLC grading assessment for IA-stage lung INMA patients. The average AUCs for this model in the internal, independent, and external test sets were 0.928, 0.837, and 0.748, respectively, indicating significant potential for predicting the new IASLC grading in early-stage lung INMA. Zhong et al [28] developed a DL model based on [^18^F]FDG PET/CT images, which performed significantly better in a large cohort than traditional PET/CT and clinical models. The AUC values for this model in the validation set, external cohort, and prospective cohort were 0.862, 0.844, and 0.851, respectively. Additionally, high-risk individuals identified with the help of this model had a better prognosis after undergoing lobectomy and systemic lymph node dissection. Thus, this model is a practical tool for identifying and improving the prognosis of high-risk clinical stage I lung INMA patients.

**Fig. 3:**
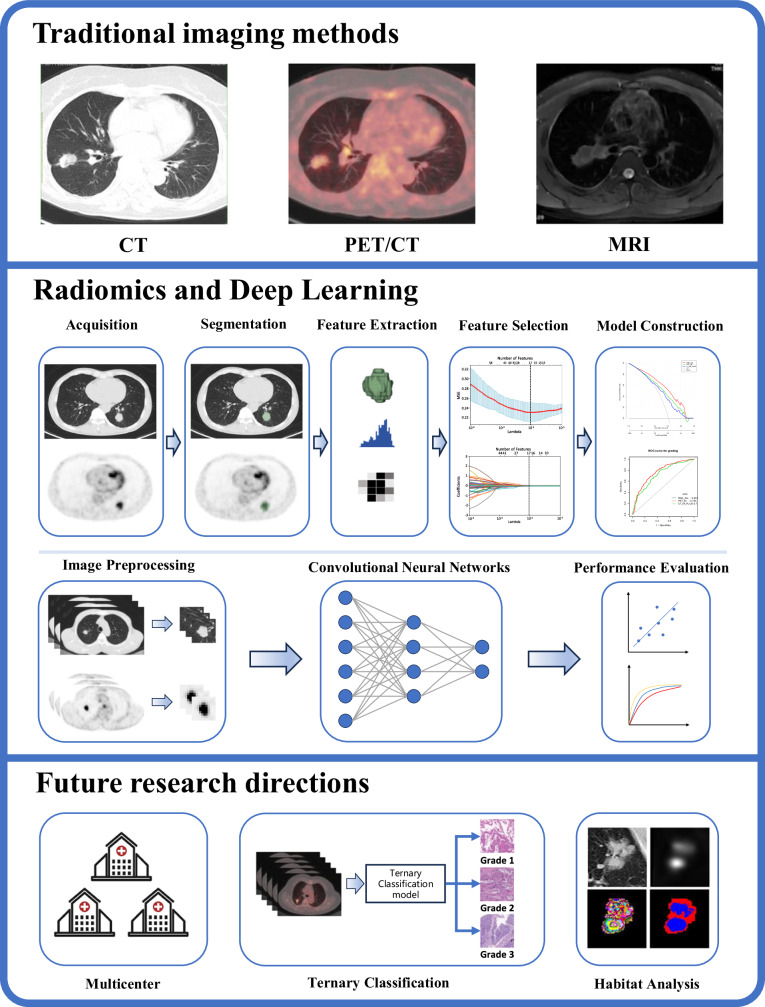
Schematic representation of non-invasive imaging methods available for predicting the new IASLC grading

Radiomics and deep learning play an important role in medical image analysis, but they still have shortcomings. These include the need to obtain large-scale and high-quality medical imaging data, the difficulty in interpreting the decision-making process of deep learning models, and the model’s generalization performance being influenced by various factors. Additionally, the new IASLC grading system classifies lung INMA into three grades. However, current studies mostly use binary classification methods to predict the tumor, such as Grade 1/2 vs. Grade 3 or Grade 1 vs. Grade 2/3, and there are no reports of ternary classification models that can predict all three tumor grades. Pan et al [81] proposed a method combining deep learning and radiomics in a study predicting the invasiveness of lung adenocarcinoma through chest CT. This method improves the performance of deep learning models through framework optimization, joint learning, and adjudication strategies. The introduced adjudication strategy resolves classification conflicts by integrating two binary classification models and one ternary classification model, thereby improving the model’s accuracy in predicting lung adenocarcinoma invasiveness. Therefore, ternary classification models may be used to improve the accuracy of radiomics and deep learning methods in predicting the new IASLC grading. Moreover, traditional radiomics methods primarily extract features based on the entire tumor lesion, assuming that the tumor is homogeneous or that tumor heterogeneity is uniformly distributed throughout the tumor [82]. However, 90% of lung INMA exhibit more than two pathological histological patterns simultaneously, with varying proportions, indicating high tumor heterogeneity [4, 83]. Habitat imaging technology divides and visualizes different sub-regions within the tumor using unsupervised clustering algorithms. Combined with traditional radiomics methods, this can significantly improve the diagnostic performance for lung cancer. The habitat imaging methods based on [^18^F]FDG PET/CT radiomics have already been used in lung disease research [84], and habitat analysis is also expected to become a useful tool for predicting IASLC grading. Schematic representation of non-invasive imaging methods available for predicting the new IASLC grading is in Fig. 3.

## Conclusion

The new IASLC grading system has been widely recognized and utilized. It significantly improves the prognosis of patients with lung INMA through effective prognostic stratification and opens up new research areas. Currently, numerous studies are exploring non-invasive imaging methods to predict the new IASLC grading. Preliminary results of these studies showed the feasibility of imaging methods for preoperative non-invasive prediction of new IASLC grading and to guide therapeutic strategies. It can provide important early risk assessment in diagnosis, contribute to personalized treatment plans in therapeutic decision-making, and facilitate optimization of monitoring and follow-up in patient management. However, these studies have certain limitations. For example, many studies are single-center and retrospective, which restricts the generalizability and reproducibility of the results. Imbalances in the number of cases and differences in the cases enrolled also affect the results of comparisons across studies. Additionally, related research is still insufficient, given the relatively recent establishment of the new IASLC grading system. Therefore, enhanced data diversity, algorithm optimization and multi-center prospective cohort studies are needed to further validate and extend previous results to improve the reliability of predictions for the IASLC grading. With the rapid development of medical imaging and innovative research methods, future studies are expected to employ new approaches, such as ternary classification and habitat analysis, to predict IASLC grading. These methods may improve the accuracy and stability of predictions, providing more precise information for personalized treatment.

In summary, the research of non-invasive imaging methods in predicting the IASLC grading has important clinical significance, which not only provides patients with better prognostic assessment tools, but also lays the foundation for the development of personalized treatment strategies. Despite the challenges, the future of non-invasive imaging methods for predicting IASLC grading remains promising. With further research and technological advances, it is anticipated that more researchers will focus on advancing this field, significantly improving the prognosis for patients with lung INMA.

## Supplementary information

ELECTRONIC SUPPLEMENTARY MATERIAL

## Data Availability

The datasets used and/or analyzed during the current study are available from the corresponding author upon reasonable request.

## Abbreviations

AAH: Atypical adenomatous hyperplasia
ADC: Apparent diffusion coefficient
AIS: Adenocarcinoma in situ
Arch: Architectural
ATS: American Thoracic Society
AUC: Area under the curve
CTR: Consolidation tumor ratio
DL: Deep learning
DWI: Diffusion-weighted imaging
ERS: European Respiratory Society
FDG: Fluorodeoxyglucose
HR: Hazard ratio
IAC: Invasive adenocarcinoma
IASLC: International Association for the Study of Lung Cancer
INMA: Invasive non-mucinous adenocarcinoma
LDCT: Low-dose CT
MTV: Metabolic tumor volume
OS: Overall survival
RFS: Recurrence-free survival
SUVmax: The maximum standardized uptake value
T1W: T1-weighted
T2CR: T2-weighted contrast ratio
T2W: T2-weighted
TLG: Total lesion glycolysis
WHO: World Health Organization
