# Supplementary material for: Application and progress of non-invasive imaging in predicting lung invasive non-mucinous adenocarcinoma under the new IASLC grading guidelines
Source: Insights Imaging. 2025 Jan 2;16:4. doi: 10.1186/s13244-024-01877-4 (PMC11695567; doi:10.1186/s13244-024-01877-4)
Supplement: Supplementary file 1 — ELECTRONIC SUPPLEMENTARY MATERIAL [file 13244_2024_1877_MOESM1_ESM.pdf]

# Application and Progress of Non-Invasive Imaging in Predicting Lung Invasive Non-Mucinous Adenocarcinoma under the New IASLC Grading Guidelines

# ELECTRONIC SUPPLEMENTARY MATERIAL

## Literature search strategy

For this narrative review, a literature search was conducted using PubMed databases and WANFANG DATA from June 2020 to June 2024. The following terms were searched in PubMed databases： (("Adenocarcinoma of Lung"[Mesh]) OR (Lung adenocarcinoma[Title/Abstract]) OR ( Pulmonary Adenocarcinoma[Title/Abstract])) AND ((grade[Title/Abstract]) OR (grading[Title/Abstract])) AND (("Positron Emission Tomography Computed Tomography"[Mesh]) OR (Computed Tomography[Title/Abstract]) OR (emission-computed tomography[Title/Abstract]) OR (PET[Title/Abstract]) OR (Positron Emission Tomography[Title/Abstract]) OR (18F-FDG PET/CT[Title/Abstract]) OR (Computed Tomography[Title/Abstract]) OR (CT[Title/Abstract]) OR (MRI[Title/Abstract])). And the following terms were searched in WANFANG DATA: ((Lung adenocarcinoma) OR (Lung invasive non-mucinous adenocarcinoma)) AND (grade) AND (CT OR PET/CT OR MRI OR PET/MR). 102 results were obtained and screened with title and abstract review. Potentially relevant articles were reviewed in full text. The articles Included must meet the following criteria: (1) The articles must be original

articles that are not reviews, editorials, abstracts, and case reports; (2) The population included in studies was primary lung INMA cases; (3) The articles used non-invasive imaging methods for research; (4) Studies based on the new IASLC grading system proposed in 2020. The final results were 11 papers. It included researches based on imaging technologies such as CT, PET/CT, MRI and radiomics methods.
